# Iran's humanitarian crisis: war, legality, and the erosion of population health

**DOI:** 10.1016/j.lanepe.2026.101656

**Published:** 2026-03-18

**Authors:** Karl Blanchet, Sultan Barakat, Bernadette Kumar, Paul Spiegel

**Affiliations:** a Geneva Centre of Humanitarian Studies, Faculty of Medicine, University of Geneva, Switzerland; b College of Public Policy, Hamad Bin Khalifa, Doha, Qatar; c Norwegian Institute of Public Health, Oslo, Norway; d Johns Hopkins Center for Humanitarian Health and Johns Hopkins Bloomberg School of Public Health, Baltimore, MD 21205, USA

On February 28, 2026, coordinated Israeli and US airstrikes hit Tehran and several other Iranian cities.^1^ The attacks followed the 12-day war of June, 2025, and formed part of Israel's longstanding strategy to prevent Iran from advancing its nuclear programme, dismantle its long-range missile capabilities, and weaken its regional alliances—particularly with Hizballah in Lebanon. By the fourth day, the conflict escalated across the Gulf, causing the closure of the Strait of Hormuz and dramatic spikes in global oil prices, underscoring the global economic reverberations of the escalation.

Yet again in the midst of a conflict, the most immediate and enduring consequences are borne by civilians. Modern aerial warfare rarely spares public health infrastructure. Hospitals, clinics, a primary school, and transport corridors have been struck or rendered inoperative, compounding the human toll. Between February 28th and March 5th 2026, casualties in Iran have already exceeded 1230 deaths, including more than 160 children when their school in the city of Minab was hit on February 28th.^2^ But the indirect consequences are likely to be far greater. Disruptions to chronic disease management, maternal and neonatal care, infectious disease control, and mental health services generate excess mortality and long-term morbidity that often surpass direct battlefield fatalities.^3^

This emergency unfolds against a backdrop of prolonged economic fragility and structural weaknesses in Iran's health and social systems as a result of decades of sanctions. Inflationary pressures, restricted procurement pathways for essential medicines, and environmental stressors such as water scarcity have already strained institutional resilience. Armed conflict amplifies these vulnerabilities and accelerates health system deterioration rather than causing temporary disruption. Although humanitarian exemptions formally exist, practical obstacles in banking, insurance, and trade frequently impede access to medicines and medical equipment. Women, children, refugees, and socioeconomically marginalised populations bear disproportionate burdens, and further widening pre-existing inequalities in access to care.

International humanitarian law (IHL) and the United Nations (UN) Charter are central to understanding both the escalation and its humanitarian consequences. The UN Charter prohibits the use of force except in cases of self-defence against an actual or imminent armed attack, or with explicit Security Council authorisation.^4^

Some analysts, including those at the European Council on Foreign Relations, have characterised the US-led strikes as lacking clear Security Council authorisation and failing to meet the Article 51 threshold for lawful self-defence.^5^ If this interpretation holds, the campaign risks weakening the normative architecture designed to prevent cycles of escalation that expose civilian populations to large-scale harm.

Regardless of the legality of resorting to force (*jus ad bellum*), the conduct of hostilities (*jus in bello*) remains governed by IHL.^6^ All parties—the USA, Israel, and Iran—are bound by the principles of distinction, proportionality, and precaution. These require differentiation between civilian and military objects, the avoidance of excessive incidental harm relative to anticipated military advantage, and the adoption of feasible measures to minimise civilian suffering.^7^ Medical personnel and facilities are protected at all times unless used for military purposes.

Reports of damage to hospitals and essential infrastructure raise grave concerns, not only in Iran but also in Gulf countries affected by Iranian strikes.^8^ Even where facilities are not directly targeted, reverberating effects—such as power outages, fuel shortages, supply chain disruption, and staff displacement—can disable dialysis services, neonatal care, oncology treatment, emergency obstetrics, and chronic disease management. Compliance with IHL demands not only avoidance of direct attacks but also anticipatory evaluation and mitigation of foreseeable systemic consequences for civilian health.

International human rights law, including the right to the highest attainable standard of health, remains applicable during armed conflict and economic crises. States must refrain from actions that foreseeably undermine access to essential services and must ensure that economic measures do not generate disproportionate humanitarian harm. Monitoring and mitigating the health effects of sanctions is therefore both a legal obligation and a public health imperative.^9^

Iran's role as host to millions of Afghan refugees adds further complexity.^10^ Refugees and undocumented migrants often face financial and administrative barriers to health care, increasing vulnerability to communicable disease, maternal mortality, and unmanaged chronic conditions. Escalation risks generating new displacement flows while straining already limited service capacity.

Women and children face heightened risks in such environments. Interruptions to reproductive health services, psychosocial care, and immunisation programmes can produce long-term generational consequences. Economic hardship and insecurity also elevate exposure to gender-based violence and mental health disorders. Addressing these vulnerabilities requires coordinated humanitarian, legal, and health system responses.

The crisis is now extending well beyond Iran to Gulf countries and potentially further afield as hostilities escalate, illustrating how the erosion of legal norms governing the use of force reverberates through health systems and affects the social determinants of wellbeing. Across the region, civilian populations face not only the immediate risks of violence but also the indirect consequences of damaged infrastructure, disrupted services, and strained public health systems. When military action proceeds without clear legal justification, and when civilian infrastructure is degraded directly or indirectly across multiple countries, the consequences manifest as excess mortality, disrupted life trajectories, and intergenerational health loss.

The global health community must respond with clarity and unity. All parties must fully comply with IHL, ensuring the protection of medical personnel, facilities, and civilian infrastructure. International actors should operationalise safeguards that preserve access to essential medicines and health supplies, even amid geopolitical tensions. Independent monitoring and accountability mechanisms must be strengthened to document and deter violations affecting civilian health.

The intersection of war, legality, and public health in Iran is not isolated; similar dynamics have unfolded in Ethiopia, Gaza, Sudan, and Ukraine. The legal architecture that protects civilian life is inseparable from global health security. When that architecture weakens—through unlawful force, disregard for proportionality, or sustained economic strangulation—the result is not only regional political instability but preventable human suffering on a population scale.

## Contributors

KB drafted the first version and SB, BK and PS finalised the article. All four authors validated the final version.

## Declaration of interests

None declared.
